# Cell-Extrinsic Effects of Tumor ER Stress Imprint Myeloid Dendritic Cells and Impair CD8^+^ T Cell Priming

**DOI:** 10.1371/journal.pone.0051845

**Published:** 2012-12-18

**Authors:** Navin R. Mahadevan, Veronika Anufreichik, Jeffrey J. Rodvold, Kevin T. Chiu, Homero Sepulveda, Maurizio Zanetti

**Affiliations:** 1 The Laboratory of Immunology, Department of Medicine and Moores Cancer Center, University of California San Diego, La Jolla, California, United States of America; 2 BD Biosciences, San Diego, California, United States of America; University Medical Center Freiburg, Germany

## Abstract

Tumor-infiltrating myeloid cells, such as dendritic cells (BMDC), are key regulators of tumor growth. However, the tumor-derived signals polarizing BMDC to a phenotype that subverts cell-mediated anti-tumor immunity have yet to be fully elucidated. Addressing this unresolved problem we show that the tumor unfolded protein response (UPR) can function in a cell-extrinsic manner via the transmission of ER stress (TERS) to BMDC. TERS-imprinted BMDC upregulate the production of pro-inflammatory, tumorigenic cytokines but also the immunosuppressive enzyme arginase. Importantly, they downregulate cross-presentation of high-affinity antigen and fail to effectively cross-prime CD8^+^ T cells, causing T cell activation without proliferation and similarly dominantly suppress cross-priming by bystander BMDC. Lastly, TERS-imprinted BMDC facilitate tumor growth *in vivo* with fewer tumor-infiltrating CD8^+^ T cells. In sum, we demonstrate that tumor-borne ER stress imprints *ab initio* BMDC to a phenotype that recapitulates several of the inflammatory/suppressive characteristics ascribed to tumor-infiltrating myeloid cells, highlighting the tumor UPR as a critical controller of anti-tumor immunity and a new target for immune modulation in cancer.

## Introduction

The tumor microenvironment and tumor cells harbor *noxae*, such as low nutrient supply, hypoxia, low extracellular pH, viruses, and defects in glycoprotein and lipid biosynthesis, which induce stress of the endoplasmic reticulum (ER) in tumor cells. Eukaryotic cells cope with ER stress by engaging a conserved set of intracellular signaling pathways known collectively as the unfolded protein response (UPR). The UPR is initiated by the protein chaperone 78 kDa glucose response protein (GRP78), which, in unstressed cells, binds to and keeps in an inactive state three transmembrane sensor/signaling molecules: IRE1α, ATF6, and PERK. Upon induction of ER stress, GRP78 disengages from the sensor/signaling molecules, thereby activating adaptive downstream UPR signaling [1], [2]. Involved in this homeostatic/regulatory cascade are two target genes *Xbp-1* and its spliced form *Xbp-1s* down stream of IRE1α, and *Chop* downstream of PERK, involved in decreasing ER protein folding load or inflammation and apoptotsis, respectively [3], [4].

UPR signaling pathways are activated in primary solid tumors of diverse histological origin, but not in peritumoral areas, and ablation of UPR elements prevents tumor initiation or significantly decreases tumor growth, survival, and angiogenesis [5], [6], [7], [8]. Thus, the UPR is recognized as a crucial cell-intrinsic survival mechanism in tumor cells [9]. While inflammation within the tumor microenvironment is associated with abnormalities in infiltrating myeloid cells, decreased immunity, and tumor progression, and a connection between inflammation and the UPR is known [10], [11], limited evidence suggests that the UPR may be a cell-extrinsic regulator of immunity, potentially affecting both the innate and adaptive cellular compartments [11], [12], [13]. Since innate and adaptive immune responses play a crucial role in anti-tumor defense and their subversion leads to tumor escape, it would be important to define the potential role of the UPR in the context of anti-tumor immunity.

Tumors coordinately regulate infiltrating myeloid cells, including macrophages and dendritic cells (DC), via cell-extrinsic mechanisms, causing polarization to a phenotype that facilitates tumor growth [14] through both inefficient priming of anti-tumor T cell responses and T cell-independent mechanisms such as promotion of angiogenesis and release of tumorigenic cytokines [15]. Although equipped with signals necessary for efficient T cell priming, tumor-infiltrating DC instead inhibit T cell proliferation [16], [17]. However, the nature of the tumor-derived signals driving myeloid DC dysregulation, which ultimately undermines anti-tumor CD8^+^ T cell immunity, has yet to be elucidated.

Recently, we reported a previously unappreciated cell-extrinsic effect of the tumor UPR on macrophages, transmissible ER stress (TERS), *i.e*., an event through which the ER stress response is communicated from tumor cells to macrophages [18]. As a result these cells become polarized to a novel pro-inflammatory/suppressive phenotype postulated to have pro-tumorigenic properties [18]. We proposed that myeloid DC could also be the target of tumor UPR-borne cell-extrinsic effects, ultimately facilitating tumor escape and growth [11]. To test this hypothesis, here we interrogated the cell-extrinsic consequences of the tumor UPR on myeloid DC, including effects on cell polarization, antigen presentation, CD8^+^ T cell cross-priming, and tumor growth *in vivo*.

## Materials and Methods

### Cell Culture and Conditioned Media (c.m.) Generation

B16.F10 and LLC tumor cells were grown in High Glucose DMEM (Cellgro) supplemented with 10% FBS (HyClone), pencillin/streptomycin/L-glutamine, NEAA, sodium pyruvate, HEPES, and 50 µM ß-ME. TRAMP-C1 (TC1) cells were grown in RPMI 1640 (Cellgro) containing the same supplements. To generate ER stress c.m., tumor cells were treated for 2 hrs with 300 nM thapsigargin (Tg, Enzo Life Sciences), washed twice with dPBS^-^ and re-supplemented with fresh cell culture medium for a further 16 hrs. Before use, ER stress c.m. was centrifuged at 2000 rpm for 10 min and passed through a 0.22 µm syringe filter.

### BMDC Generation

Bone marrow cells from C57BL/6 mice were flushed from femurs and tibias in cold, unsupplemented RPMI 1640. After red blood cell lysis, bone marrow cells were cultured for 6 days in complete RPMI medium containing 10% v/v supernatant from mGMCSF-producing hybridoma cells (GCM, courtesy of Dr. R. Steinman). Every two days, cells were washed and re-supplemented with complete RPMI containing 10% GCM, yielding >85% immature CD11b^+^/CD11c^+^ myeloid BMDC on day 6.

### BMDC Cross Presentation and CD8^+^ T cell Co-culture

BMDC were first exposed to tumor ER-stress c.m. or control media for 8 hrs after which heat-treated OVA (1 mg/mL) (Sigma) was added to cultures for a further 16 hrs. CD8^+^ T cells were negatively selected (StemCell Tech) from a spleen/lymph node cell suspension from OT-I mice (kindly provided by Drs. S. Schoenberger; La Jolla Institute for Allergy and Immunology), J. Chang (UCSD), and N. Gascoigne (The Scripps Research Institute) and the yield of transgenic cells was determined by enumeration of Vα2^+^/CD8^+^ T cells by flow cytometry, which was >90%. 2.5×10^5^ Vα2^+^/CD8^+^ T cells were then co-cultured with 10^5^ BMDC for 96 hrs. In some cases, negatively selected CD8^+^ T cells were labeled with 0.5 µM CFDA-SE (Invitrogen) before co-culture. In the exogenous antigen rescue experiments, TERS-imprinted BMDC were pulsed with SIINFEKL peptide (1 µg/mL) for 4 hrs at 37°C prior to co-culture. In the arginase inhibition experiments, 2 mM L-arginine (EMD Chemicals) or 10 mM L-norvaline (EMD Chemicals) were added to cell culture medium prior to addition of BMDC and OT-I T cells. In the anergy experiments, recombinant mouse (rm)IL-2 (R&D Systems) was added (30 U/mL or 100 U/mL) to cell culture medium prior to addition of BMDC and OT-I T cells. In the restimulation experiments, T cells were recovered from 96-hr co-cultures using Lympholyte M (Cedar Lane), washed and cultured in complete RPMI for an additional 48 hrs. T cells were restimulated or not with SIINFEKL (1 µg/mL)-pulsed BMDC, with or without the addition of rmIL-2 (30 U/mL) to the co-culture for an additional 48 hrs.

### Flow Cytometry

Single cell suspensions of BMDC, T cells or *in vivo* tumor samples, were stained with fluorophore-conjugated anti-CD86 (BD Biosciences, clone GL1), anti-CD80 (BD Biosciences, clone 16-10-A1), anti-CD40 (BD Biosciences, clone 3/23), anti-CD11b (eBioscience, clone M1/70), anti-CD11c (eBioscience, clone N418), anti-H2k^b^ (BD Biosciences, clone AF6-88.5), anti-IA^b^ (BD Biosciences, clone AF6-120.1), anti-K^b^-SIINFEKL (eBioscience, clone ebio25.D1.16), anti-PDL-1 (BD Biosciences, clone MIH5), anti-CD8α (eBioscience, clone Ly-2), anti-Vα2 (BD Biosciences, clone B20.1), anti-CD69 (BD Biosciences, clone H12F3), anti-CD25 (BD Biosciences, clone PC61.5), anti-CD62L (BD Biosciences, clone MEL14), anti-CD44 (BD Biosciences, clone IM7), anti-IFN-γ (BD Biosciences, clone XMG1.2), anti-PD-1 (BD Biosciences, clone RMP1–30), anti-CD28 (BD Biosciences, clone 37.51), anti-LAG3 (BD Biosciences, clone C9B7W), and anti-FOXP3 (BD Biosciences, clone MF23) antibodies, or appropriate isotype controls. Viability was determined by 7-AAD exclusion. Data were acquired on a FACSCalibur flow cytometer (Becton Dickinson) and analyzed using CellQuest Pro (BD Biosciences) and FlowJo software (Tree Star).

### RT-qPCR

mRNA was isolated from BMDC or T cells after Lympholyte-M (Cedar Lane) purificationusing the RNA II Nucleospin Kit (Macherey-Nagel). Concentration and purity of RNA were determined by analysis on a NanoDrop spectrophotometer (ThermoScientific). cDNA was obtained using the High Capacity cDNASynthesis kit (Life Technologies/Applied Biosystems), and RT-qPCR was performed on an ABI StepOne system using TaqMan reagents for 50 cycles using universal cycling conditions. Target gene expression was normalized to *β-actin*, and analyzed using the –ΔΔCt relative quantification method. Validated FAM-labeled mouse *Il-23p19(a)*, *Il-6*, *Tnf*, *Il-17a*, *Il-10*, *Foxp3*, *Ddit3* (Chop), *Hspa5* (Grp78), *Arg1*, and VIC-labeled mouse *β-actin* TaqMan primer/probe sets (Life Technologies/Applied Biosystems) were used. FAM-labeled qPCR probe/primer sets specific for the spliced form of mouse *Xbp-1* was obtained from Integrated DNA Technologies.

### BD® Cytometric Bead Array Assay

BD® CBA Flex set assays were used to measure mouse IL-6, IL-23, TNF-α, MIP-1α, MIP-1β, and MCP-1, IL-2, IL-10, IL-17, and TGF-ß (BD Biosciences). Following acquisition of sample data using a BD® FACSArray bioanalyzer flow cytometer, the results were expressed in graphical and tabular formats using the FCAP Array Software.

### Western Blot

BMDC were lysed using a RIPA lysis buffer system (Santa Cruz Biotechnology). Lysates were reduced with ß-mercaptoethanol in loading buffer and then heated at 95°C for 5 min. Samples were normalized by protein concentration determined by NanoDrop, and separated by electrophoresis on a 4–20% SDS-PAGE gel (Bio-Rad). Following electrophoresis, the fractionated proteins were transferred to PVDF membranes (Bio-Rad) using a wet transfer system (Life Technologies). Blots were then blocked with 5% non-fat milk powder in Tris-buffered saline with Tween buffer. PVDF membranes were then incubated with a rabbit antibody to mouse GRP78 (Abgent) overnight at 4°C. PVDF membranes were subsequently incubated with a HRP-conjugated goat antibody to rabbit Ig (Jackson ImmunoResearch). A HRP-conjugated rabbit antibody to β-actin was also used as a loading control. Bands were visualized using via an enhanced chemiluminescence system (Thermo Scientific).

### Arginase Activity Assay

Cells lysates were prepared in 50 µL of lysis buffer (Triton X-100 0.5%, HEPES 50 mmol/L, NaCl 150 mM/L, sodium orthovandate 1 mM/L, PMSF 2 mM/L and a protease inhibitor cocktail 75 µg/mL), and centrifuged at 16000 xg for 16 min at 4°C. A BCA assay (Pierce 23227) was performed to determine the cell lysates’ protein concentrations. Cell lysates (5 µg) were added to 25 µL of Tris-HCl (50 mM; pH 7.4) containing 10 mM MnCl_2_. The mixture was heated at 55–60°C for 10 min to activate arginase. A sample containing Triton X-100 served as the blank. 150 µL of Tris-HCl (100 mM) and 50 µL of L-Arg were added to each sample and incubated at 37°C for 20 min to begin the reaction. The reaction was stopped by the addition of 0.72 M HCl (volume equivalent to that of sample) and by centrifugation at 5000 rpm for 5 min at room temperature. Aliquots (120 µL) of the supernatants and the ladder sample (50 mM, 5 mM, 500 µM, 50 µM, 5 µM and 500 nM of L-ornithine) were incubated with 6% ninhydrin (264 µL) at 95°C for 1 hr to determine the amount of reaction product, L-ornithine, using a colorimetric assay. The supernatants were then centrifuged at 5000 rpm for 5 min at room temperature and aliquots (95 µL) were transferred to a 96-well Costar plate and read at A510 nm. Results were expressed as millimoles of L-ornithine.

### *In vivo* Tumor Studies

BMDC were exposed to TERS c.m. or (unstressed) tumor cell c.m. for 24 hrs. Tumor cells and BMDC were harvested and washed twice with dPBS^-^. Tumor cells were admixed with BMDC at a 3∶1 ratio (3×10^4^ B16.F10∶10^4^ BMDC or 3×10^6^ TC1.OVA:10^6^ BMDC) and injected s.c. into 8–10 week-old C57BL/6 mice. Experiments using TC1.OVA cells were conducted using male mice only. Tumor growth was measured by taking two-dimensional caliper measurements starting 4 days after injection until tumors reached ≥20 mm in one dimension, at which time the mice were sacrificed in accordance with UCSD animal welfare standards. Tumor size was expressed as volume (mm^3^) using the ellipsoid volume formula, V = ½ (H x W^2^). For CD8^+^ T cell quantification experiments, tumors and draining lymph nodes were surgically excised and mechanically dispersed through 40 µm filter into cell suspensions, and live cells (7AAD^-^) interrogated for CD8 expression by flow cytometry. This study was carried out in strict accordance with the recommendations in the Guide for the Care and Use of Laboratory Animals of the National Institutes of Health under protocol No. S00022 and S00023 approved by the UCSD Institutional Animal Care and Use Committee (IACUC).

#### Animal welfare

Mice animals unable to self-feed because of the tumor, were inspected and euthanized. Mice inoculated with tumor were sacrificed when tumor mass reached 2 cm in diameter in any dimension. In case of infection or ulceration mice were euthanized per UCSD IACUC policy. Mice were euthanized by the administration of C02 followed by cervical dislocation.

## Results

### Tumor ER Stress is Transmitted to Myeloid Dendritic Cells

**Figure 1:**
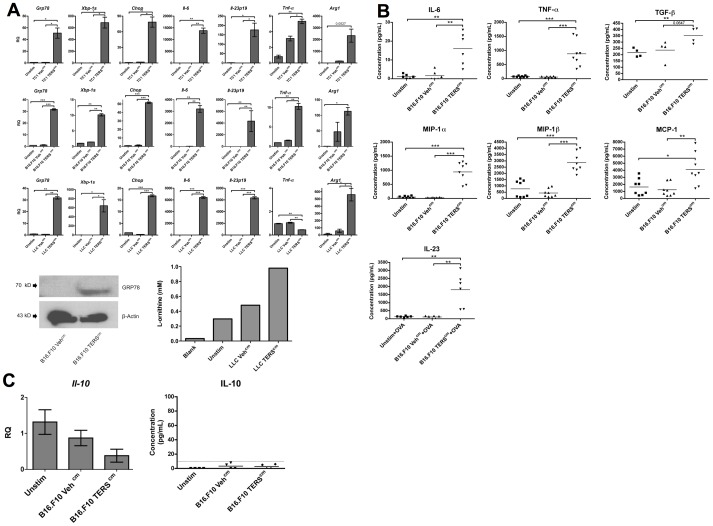
TERS-imprinted BMDC upregulate elements of the UPR signaling pathways and produce pro-inflammatory cytokines. BMDC were cultured for 24 hrs in TERS^cm^ or Veh^cm^ from the tumor cell lines indicated, or media alone (Unstim). (**A**) RNA was isolated from BMDC and analyzed by RT-qPCR for UPR activation and proinflammatory cytokine gene transcription. Columns indicate fold increase in transcript level (RQ) of each treatment group. An Unstim control was set arbitrarily to 1. Error bars represent SEM of two biological replicates and are representative of six independent experiments. *P<0.05, **P<0.01, ***P<0.001, unpaired, two-tailed *t* test. BMDC cell lysates were analyzed for GRP78 expression by Western blot as indicated in Materials and Methods. Arginase activity was determined through the hydrolysis of L-arginine to L-ornithine. The amount of L-ornithine produced was determined using a colorimetric assay with ninhydrin, and was quantified using a ladder of known L-ornithine concentrations. The results are representative of two independent experiments. (**B**) Supernatants from BMDC in (A) were analyzed by cytometric bead array for presence of the indicated cytokines. *P<0.05, **P<0.01, ***P<0.001, unpaired, two-tailed *t* test. (**C**) RNA was isolated from BMDC and analyzed by RT-qPCR for *Il-10* transcription. Columns indicate fold increase in transcript level (RQ) of each treatment group. An Unstim control was set arbitrarily to 1. Error bars represent SEM of four biological replicates pooled from two independent experiments. BMDC supernatants were interrogated for IL-10; the dotted line indicates the threshold of detection.

The cell-extrinsic effects of tumor UPR were assessed by culturing bone marrow-derived dendritic cells (BMDC) in the conditioned medium (c.m.) of ER-stressed murine tumor cells (prostate, TRAMP-C1 or TC1; melanoma, B16.F10; and Lewis lung carcinoma, LLC). Under these conditions, BMDC mounted a global ER stress response, as evinced by the upregulation of the master UPR regulator GRP78, and two downstream UPR effectors, *Xbp-1s*, and *Chop* (Fig. 1A). Tumor ER stress c.m. -imprinted (TERS-imprinted) BMDC also upregulated the transcription of the pro-inflammatory, pro-tumorigenic cytokines *Il-6*, *Il-23p19*, and, in two of three cell lines, *Tnf-α* (Fig. 1A) [19], [20], [21]. Congruently, we detected increased secretion of IL-6, IL-23, TNF-α, and the cytokines/chemokines TGF- ß, MIP-1α, MIP-1ß, and MCP-1 (Fig. 1B). No increased IL-10 transcription or secretion was detected (Fig. 1C). Importantly, TERS-imprinted BMDC also upregulated the transcription and functional activity of Arginase 1 (Fig. 1A), a known suppressor of T cell function [22]. Taken together, these findings suggest that, similar to macrophages [18], BMDC are a susceptible target of TERS, through which they assume a pro-inflammatory/suppressive phenotype.

### Activation and Maturation of BMDC Exposed to TERS

**Figure 2:**
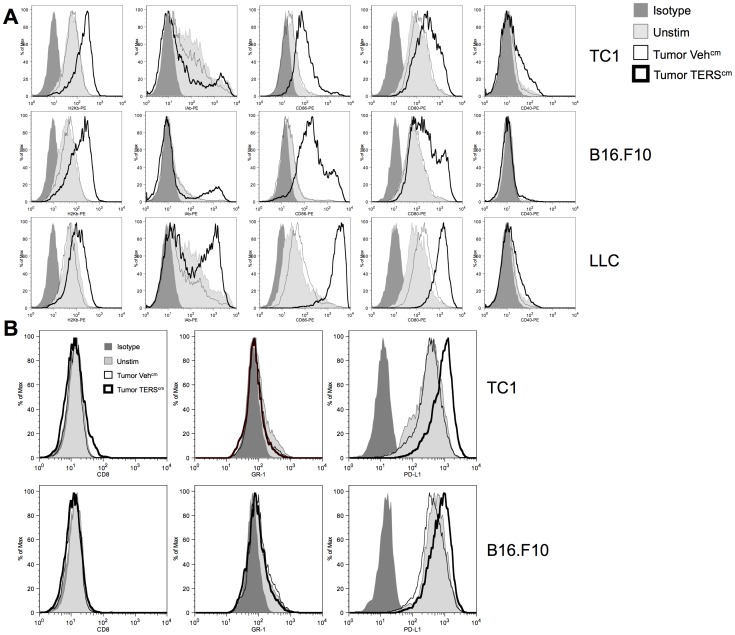
TERS-imprinted BMDC polarize to an activated, mature immunephenotype. BMDC were cultured for 24 h in TERS^cm^ or Veh^cm^ from the tumor cell lines indicated, or media alone (Unstim), and interrogated for the cell-surface expression of the indicated molecules by flow cytometry. Results are representative of at least three independent experiments.

TERS-imprinted BMDC change morphology, acquiring characteristics of activated, mature myeloid DC, including increased cell size and elongated dendrites (Fig. S1). We confirmed that TERS-imprinted BMDC undergo activation and maturation, as they upregulate expression of MHC Class I and Class II, and the costimulatory molecules CD86, CD80, and, to a lesser extent, CD40 (Fig. 2A). These cells are CD8α^ -^ (Fig. 2B), confirming their myeloid origin. GR-1, which is expressed at low levels in immature BMDC, was not upregulated by exposure to TERS (Fig. 2B), distinguishing their phenotype from that of myeloid-derived suppressor cells (MDSC), a class of myeloid cells that accumulate in the tumor microenvironment [14], [23]. Additionally, TERS-imprinted BMDC only slightly increased the expression of PD-L1 (B7H1), the ligand for the T cell immunoinhibitory PD-1 receptor [24], above constitutive levels (Fig. 2B).

### Impaired Cross-presentation and Cross-priming of CD8^+^ T cells by TERS-imprinted BMDC

**Figure 3:**
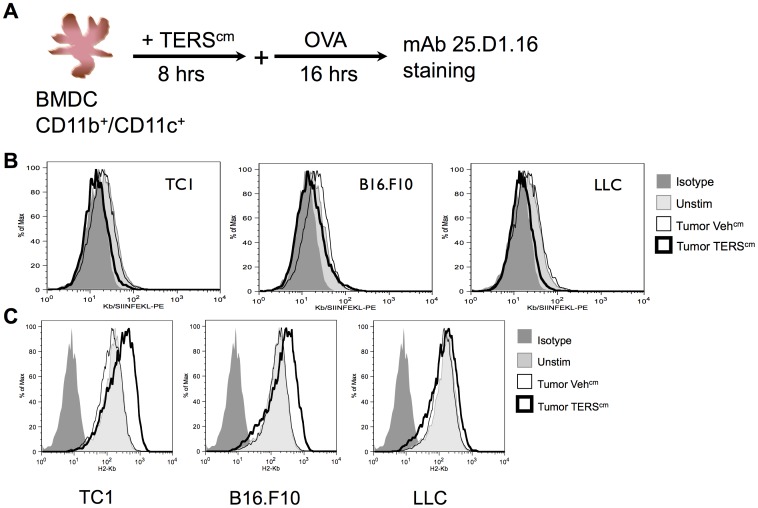
Impaired cross-presentation by TERS-imprinted BMDC. (**A**) Schematic of the cross-presentation assay. BMDC were cultured in TERS^cm^ or Veh^cm^ from tumor cell lines, or media alone (Unstim), for 8 hrs after which OVA (1 mg/mL) was added directly to cultures for a further 16 hrs period. (**B**) Cross-presentation of the SIINFEKL/H2-K^b^ complex was monitored using the 25.D1.16 antibody by flow cytometry. Results are representative of three independent experiments. (**C**) H2-K^b^ expression was measured by flow cytometry. Results are representative of four independent experiments.

Next, we explored the antigen presentation capacity of TERS-treated BMDC, focusing on cross-presentation as this mode of antigen presentation may be crucial in presenting exogenous tumor antigen to CD8^+^ T cells [25], [26], [27]. We used a system in which BMDC fed soluble ovalbumin (OVA) cross-present the SIINFEKL peptide complexed to the H2-K^b^ molecule, which we detected using the monoclonal antibody, 25.D1.16 [28] (Fig. 3A). We found that TERS-imprinted BMDC have a reduced capacity to cross-present antigen (Fig. 3B), notwithstanding the fact that the surface expression of MHC Class I molecules remained constant or even increased over that of OVA-fed control BMDC (Fig. 3C).

**Figure 4:**
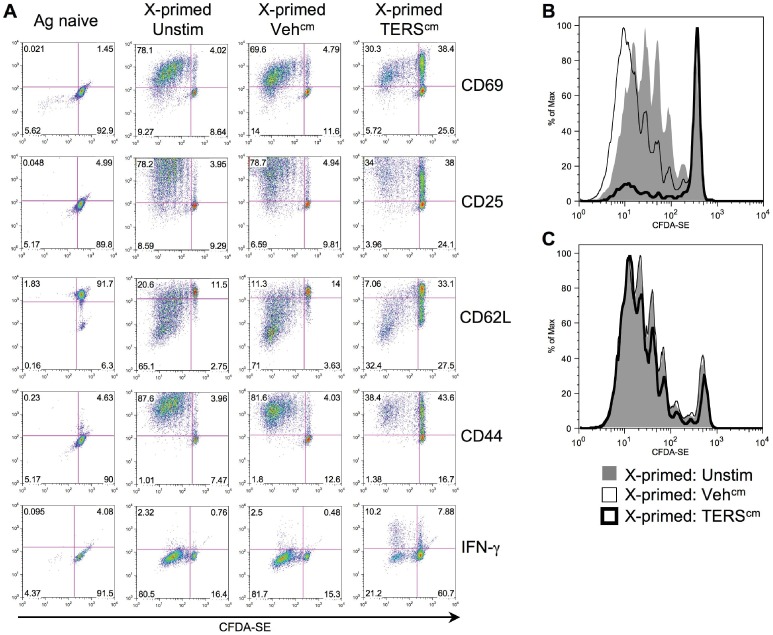
CD8^+^ T cells cross-primed by TERS-imprinted BMDC become activated but do not proliferate. Cross-presenting BMDC were prepared as in Fig. 3A. Unstimulated BMDC not fed OVA (Ag naïve) were used as a control. BMDC were then co-cultured with CFDA-SE-labeled CD8^+^ OT-I transgenic T cells. After 96 hrs co-culture, CD8^+^ T cells were interrogated for (**A**) expression of cell-surface activation markers, and (**B**) proliferation (CFDA-SE dilution) by flow cytometry. Results are representative of eight independent experiments. (**C**) CD8^+^ T cells were cultured in TERS^cm^ or Veh^cm^ from B16.F10 tumor cells, or media alone (Unstim) for 24 hrs. The CD8^+^ T cells were then labeled with CFDA-SE and incubated with OVA cross-presenting BMDC. After 96 hrs co-culture, CD8^+^ T cells were interrogated for proliferation (CFDA-SE dilution) by flow cytometry.

We then investigated the ability of TERS-imprinted BMDC to cross-prime naïve CD8^+^ T cells from OT-I mice, which express a transgenic T cell receptor (TCR) specific for the SIINFEKL/H2-K^b^ complex [29]. When fed OVA, unstimulated BMDC or BMDC treated with c.m. from unstressed tumor cells (Veh^cm^) efficiently induced OT-I CD8^+^ T cell activation and proliferation, as demonstrated by conversion to an activated CD69^+^/CD25^+^/CD62L^lo^/CD44^+^ phenotype (Fig. 4A) and by 5-(and-6)-carboxyfluorescein diacetate, succinimidyl ester (CFDA-SE) dilution (Fig. 4B), respectively. In contrast, OT-I CD8^+^ T cells cross-primed by OVA-fed, TERS-imprinted BMDC, while activated, proliferated poorly, resulting in a decreased percentage of activated, dividing T cells (Fig. 4A–B). As expected, the co-culture of OT-I T cells with untreated or TERS-imprinted BMDC without antigen, did not result in activation or proliferation (Fig 4A and Fig. S2). Pretreatment of naïve OT-I CD8^+^ T cells with TERS c.m. did not affect their ability to proliferate upon subsequent cross-priming (Fig. 4C). The proliferation defect was not associated with PD-1 upregulation on T cells (Fig. S3).

**Figure 5:**
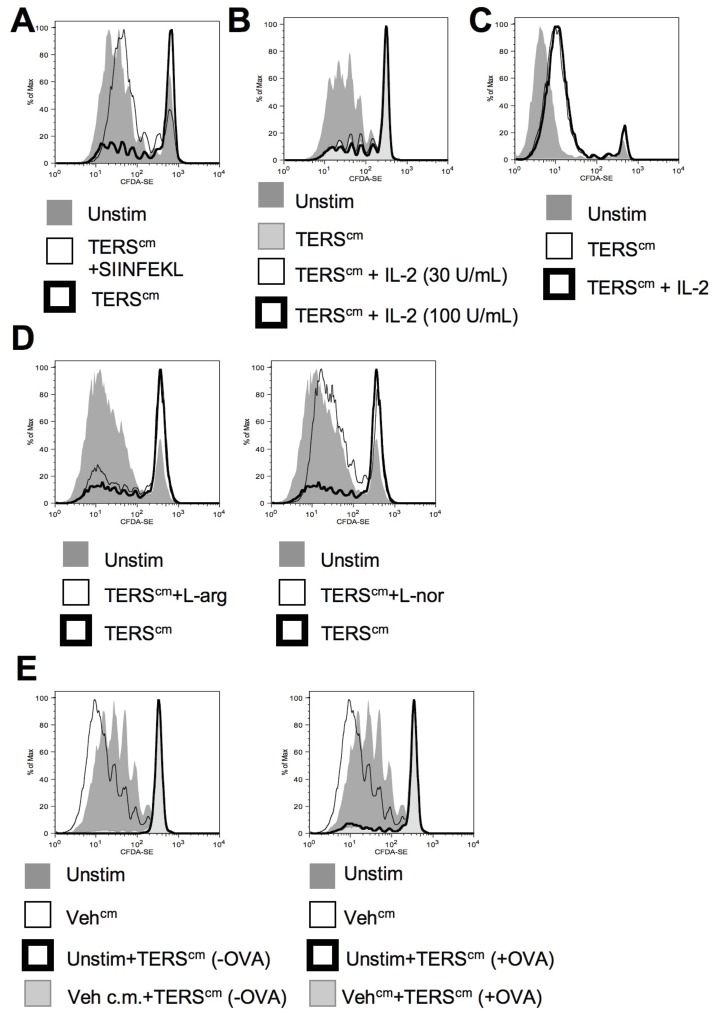
The proliferation-refractory phenotype of CD8^+^ T cells cross-primed TERS-imprinted BMDC can be rescued by excess antigen or L-norvaline, but not by addition of IL-2. BMDC were co-cultured with OT-I CD8^+^ T cells as in Fig. 4. (**A**) CD8^+^ OT-I T cells were co-cultured with SIINFEKL-pulsed TERS-imprinted BMDC and CD8^+^ T cell proliferation was measured by CFDA-SE dilution. Results are representative of four independent experiments. (**B**) Recombinant mouse IL-2 was added at 30 or 100 U/mL to the co-cultures as indicated, and CD8^+^ T cell proliferation was measured by CFDA-SE dilution. Results are representative of two independent experiments. (**C**) After 4-day co-culture, CFDA-SE-labeled CD8^+^ T cells were recovered and rested for 2 days before restimulation with SIINFEKL-pulsed BMDC, with or without exogenous rmIL-2 (30 U/mL). CD8^+^ T cell proliferation was measured by CFDA-SE dilution. Results are representative of two independent experiments. (**D**) L-arginine (L-arg, 2 mM) or L-norvaline (L-nor, 10 mM) was added to co-cultures and CD8^+^ T cell proliferation was measured by CFDA-SE dilution. Results are representative of four independent experiments. (**E**) BMDC with (+) and without (−) OVA were co-cultured with CFDA-SE-labeled CD8^+^ OT-I T cells at a 1∶1∶2.5 ratio as above and T cell proliferation was measured by CFDA-SE dilution. Results are representative of four independent experiments.

We reasoned that insufficient antigen presentation may contribute to the impaired proliferation observed in CD8^+^ T cells. Addition of exogenous peptide antigen (1 µg/mL) rescued T cell proliferation (Fig. 5A). Because a proliferation-refractory phenotype could reflect T cell anergy, and this is classically rescued by addition of exogenous IL-2 [30], we interrogated the effect of adding exogenous IL-2 in our system. Exogenous IL-2 added during cross-priming failed to rescue OT-I T cell proliferation (Fig. 5B). Removal from the co-culture with TERS-imprinted BMDC partially restored T cell proliferation, although with fewer cell divisions than control (Fig. S4), suggesting that the proliferative defect requires cell-cell contact. Exogenous IL-2 added to CD8^+^ T cells during antigen restimulation after 48-hour rest, a classical method to disclose CD4^+^ T cell anergy [30], failed to correct the proliferative lag (Fig. 5C), suggesting that the proliferation-refractory CD8^+^ T cells generated here do not fulfill the classical criteria of anergy.

The proliferative defect was reminiscent of that observed in T cells deprived of arginine [16]. Because TERS-imprinted BMDC upregulate arginase (*Arg1*) expression (Fig. 1A), we probed its contribution to the T cell cross-priming defect. Addition of exogenous L-arginine to the co-culture did not improve T cell proliferation (Fig. 5D). In contrast, addition of L-norvaline, a competitive inhibitor of arginase, rescued in great part (80%) T cell proliferation (Fig. 5D). Taken together, these results suggest that tumor UPR-mediated BMDC-derived arginase activity also contributes to the T cell proliferative defect observed.

We next investigated whether TERS-imprinted BMDC could exert dominant suppression over cross-priming by normal bystander BMDC. When TERS-imprinted BMDC, with or without OVA, were added to co-cultures of OT-I T cells and OVA-fed control BMDC, T cell proliferation was again suppressed (Fig. 5E), suggesting TERS-imprinted BMDC rapidly suppress the cross-priming capability of normal BMDC. Surprisingly, the addition of L-norvaline did not rescue T cell proliferation caused by dominant suppression (Fig. S5). Thus, not only can TERS-imprinted BMDC directly inhibit CD8^+^ T cell proliferation via cross-priming, but they can also suppress T cell cross-priming by bystander BMDC in a TCR-independent, arginase-independent manner.

**Figure 6:**
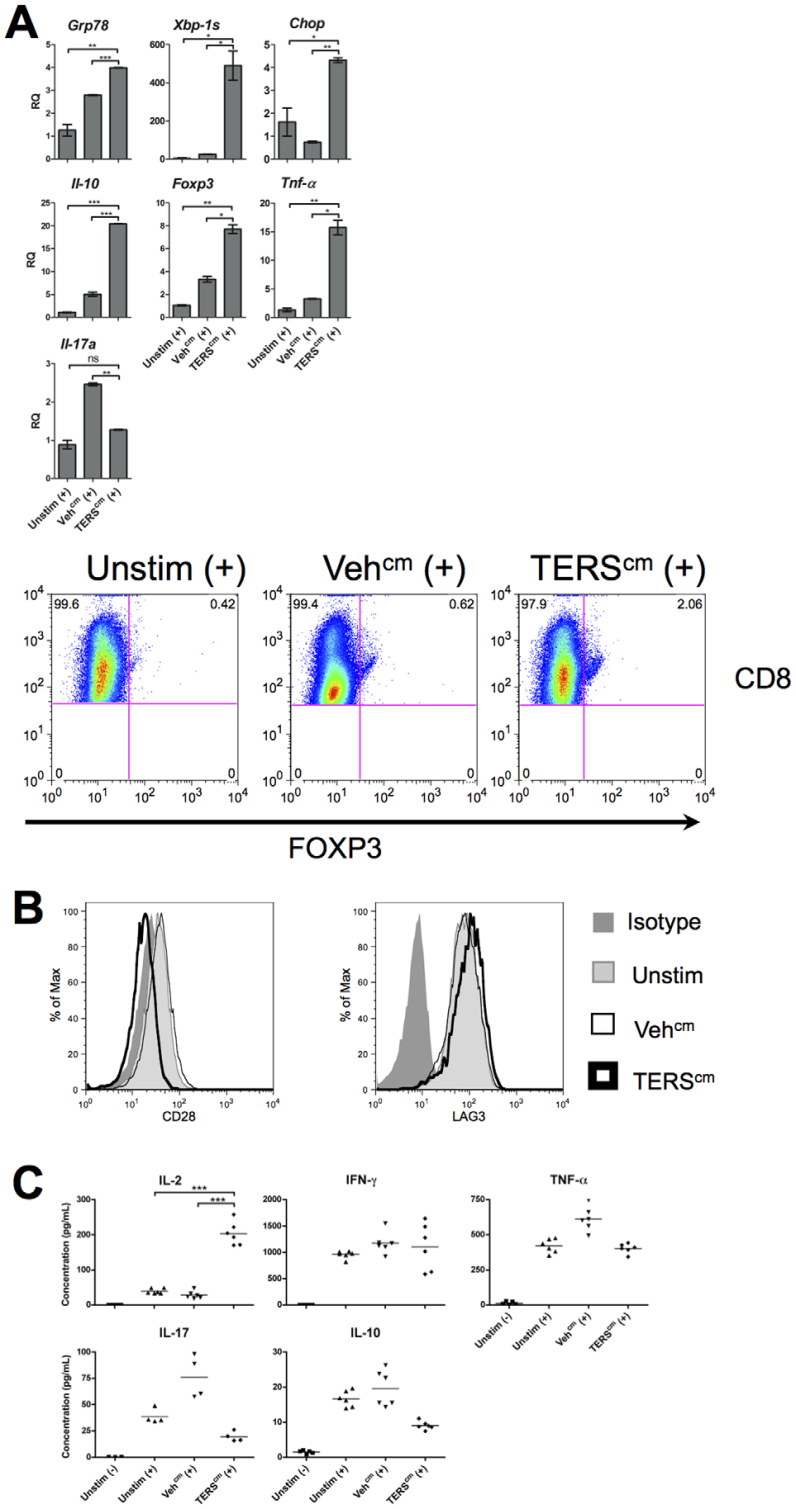
Transcriptional and phenotypic analysis of CD8 T cells cross-primed by TERS-imprinted BMDC. (**A**) After 96-hr co-culture, CFDA-SE-labeled CD8^+^ T cells were purified, the mRNA isolated and analyzed by RT-qPCR for transcription levels of the indicated genes. Columns indicate fold increase in transcript level (RQ) of each treatment group. An Unstim (+) control was set arbitrarily to 1. Error bars represent SEM of two biological replicates and are representative of four independent experiments. *P<0.05, **P<0.01, ***P<0.001, ns = not significant, unpaired, two-tailed *t* test. FOXP3 expression was interrogated by intracellular flow cytometry. (**B**) After 96 hr co-culture, CD8^+^ T cells were interrogated for CD28 and LAG3 surface expression by flow cytometry. Results are representative of three independent experiments. (**C**) Supernatants from 96 hr co-cultures were interrogated for the presence of cytokines using the BD® Cytometric Bead Array assay. Results are pooled from two independent experiments.

Initial lineage analysis of CD8^+^ T cells cross-primed by TERS-imprinted BMDC showed transcriptional upregulation of the cytokines *Il-10* and *Tnf-*α but not *Il-17* (Fig. 6A). Upregulation of FOXP3 and downregulation of the costimulatory molecule CD28 (Fig. 6A and B) were also observed. LAG3, a negative costimulatory molecule [31] found on tumor-infiltrating T cells [32], was slightly upregulated (Fig. 6B). When we analyzed the 96-hour TERS-imprinted BMDC:T cell co-culture supernatant, we observed increased secretion of IL-2 but no elevation of IL-10, IL-17, IFN-γ,or TNF-α above controls (Fig. 6C). Taken together, this suggests that CD8^+^ T cells cross-primed by TERS-imprinted BMDC display an uncommitted phenotype with potential suppressive characteristics (*Foxp3* and *Il-10* upregulation, and CD28 downregulation) [33], [34]. Surprisingly, CD8^+^ T cells cross-primed by TERS-imprinted BMDC for four days also demonstrated disproportionately high splicing of *Xbp-1* compared to the modest upregulation of other UPR elements (Fig. 6A).

### TERS-imprinted BMDC Facilitate Tumor Growth *in vivo*

**Figure 7:**
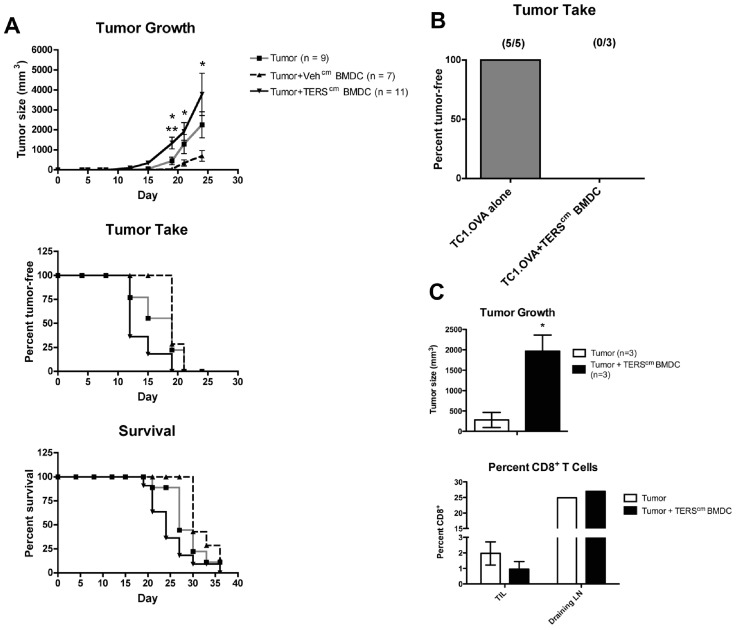
TERS-imprinted BMDC facilitate tumor growth *in vivo*. (**A**) BMDC were cultured in TERS^cm^ or Veh^cm^ from B16.F10 tumor cells for 24 hrs and admixed with B16.F10 cells at a 1∶3 ratio (10^4^ BMDC:3×10^4^ B16.F10). Mixtures or B16.F10 cells alone (3×10^4^) were injected s.c. into the flanks of C57BL/6 mice and growth monitored by caliper measurement. Tumor size was expressed as volume (mm^3^). Error bars represent SEM of tumor size measurements pooled from all animals in the indicated experimental group. Statistical comparison was made between the following groups: [Tumor+TERS^cm^ BMDC] and Tumor alone (top symbols) or [Tumor+Veh^cm^ BMDC] (bottom symbols) on day 19. All other indicated comparisons were made between [Tumor+TERS^cm^ BMDC] and [Tumor+Veh^cm^ BMDC] groups. *P<0.05, **P<0.01, unpaired, two-tailed *t* test. (**B**) BMDC were cultured in TERS^cm^ from TC1 tumor cells for 24 hrs and admixed with TC1.OVA cells at a 1∶3 ratio (10^6^ BMDC:3×10^6^ TC1.OVA). Mixtures or TC1.OVA cells alone (3×10^6^) were injected s.c. into the flanks of male C57BL/6 mice and tumor growth monitored by caliper measurement for 22 days. (**C**) B16.F10 tumor cells and TERS-imprinted BMDC were admixed as in (A) and injected s.c. into the medial thigh of C57BL/6 mice. Tumors were excised on day 14, measured by caliper (upper panel), and the percentage of tumor-infiltrating CD8^+^ T lymphocytes (TIL) quantified by flow cytometry. The percentage of CD8^+^ T lymphocytes was similarly determined in pooled draining inguinal lymph nodes (LN).

To test the role of TERS-imprinted BMDC in facilitating tumor growth *in vivo*, C57BL/6 mice were inoculated subcutaneously with B16.F10 tumor cells admixed with TERS-imprinted BMDC according to [35]. Mice so injected displayed accelerated tumor growth, earlier tumor initiation, and decreased survival as compared to mice receiving B16.F10 tumor cells admixed with control BMDC, or tumor cells alone (Fig. 7A), suggesting that BMDC polarized by ER-stressed tumor cells facilitate tumor growth *in vivo*. To specifically implicate dysfunctional anti-tumor T cell immunity as a mechanism of immune escape, we utilized TC1.OVA prostate cancer cells that constitutively express OVA, which functions as a tumor rejection antigen [36]. Whereas no tumors grew in mice inoculated with TC1.OVA cells alone, transient tumor growth, peaking at 6–10 days post-injection, occurred in mice inoculated with TC1.OVA cells admixed with TERS-imprinted BMDC (Fig. 7B).

We next interrogated the effect of TERS-imprinted BMDC on the number of tumor-infiltrating host CD8^+^ T lymphocytes (TIL) using the B16.F10 admixture model. As expected, we found that B16.F10 tumor cells admixed with TERS-imprinted BMDC grew larger than control B16.F10 tumors by day 14 (Fig. 7C, upper panel). Notably, B16.F10 tumors seeded with TERS-imprinted BMDC contained about half the percentage of CD8^+^ TIL as compared with control B16.F10 tumors (Fig. 7C, lower panel). Interestingly, while we found a decreased number of TIL in tumors, we found no difference in the draining lymph nodes, implying the local nature of this phenomenon. These results indicate that TERS-imprinted BMDC can inhibit T cell immunity *in vivo*, thus promoting tumor growth, even facilitating the take of immunogenic tumor cells.

## Discussion

The cell-intrinsic effects of the UPR on tumor survival [37], angiogenesis [38], [39], genomic instability [40], and the metabolism of cancer cells [41], are well understood. On the other hand, the cell-extrinsic effects of the tumor UPR remain poorly elucidated [11]. In this work we have shown that the tumor UPR is transmissible to myeloid DC, imprinting them with an activated phenotype associated with the secretion of pro-inflammatory, tumorigenic cytokines, but also activation of arginase, an enzyme with T cell suppressive function [16], [42]. Importantly, we also show that the transmission of the UPR to myeloid DC results in the impairment of their ability to cross-present antigen and cross-prime CD8^+^ T cells, yielding T cells with severely impaired proliferative capacity. Finally we show that TERS-imprinted myeloid DC co-injected with tumor cells into naïve immunocompetent mice promote tumor take, accelerate tumor growth, and decrease the number of CD8^+^ tumor-infiltrating lymphocytes, even promoting transient outgrowth of immunogenic tumor cells. Together these findings suggest that transmissible ER stress plays an important regulatory role at the tumor/immune interface through polarization of myeloid DC to a phenotype that ultimately hinders adaptive anti-tumor immunity. Notably, the data presented here show that tumor UPR-borne cell-extrinsic signals can recapitulate *ab initio* the activated/suppressive phenotype observed in tumor-infiltrating myeloid DC in several *in vivo* systems [16], [17].

Tolerogenic DC have been described in various systems [43], and have been defined as steady-state, immature cells able to present antigen [44]. Here, instead we show that TERS-imprinted BMDC are phenotypically mature, upregulate costimulatory molecules, and have diminished cross-presentation capacity. Defects in cross-presentation could originate from an altered MHC Class I immunopeptidome [45] as a result of transmissible ER stress. For instance, ER stress could induce a transcriptional down-regulation of the ER chaperone tapasin [46], which is important for the loading and stabilization of high affinity peptides to the MHC I molecule in the ER prior to their export to the cell surface. Such a mechanism has been reported in human cytomegalovirus-infected cells, which undergo ER stress [47], and in cancer cells after treatment with the histone deacetylase inhibitor trichostatin A, which also activates UPR genes [48]. A second possibility would be a disruption of the MHC I/peptide complex as demonstrated in mouse thymoma cells under ER stress, which have a decreased presentation of high affinity ovalbumin peptide on MHC Class I [49]. A third possibility would be that BMDC imprinted by TERS could directly disrupt the binding of MHC Class I/peptide complexes to T cells, for instance through nitration of tyrosines in the TCR as demonstrated for more conventional MDSC [50].

Ligation of the T cell receptor in the presence of costimulatory signals is required for T cell activation and, in converse, ligation in their absence leads T cells to become unresponsive [51], a condition known as anergy [52]. The data presented here show that the unresponsiveness of CD8 T cells cross-primed by TERS-imprinted myeloid DC fits, paradoxically, a scenario where costimulatory signals are provided but the display of MHC I/peptide complexes is diminished (Fig. 2). Our findings place into context previous work describing tumor associated mature myeloid regulatory DC [16], [17] by suggesting that these cells could be the result of cell-extrinsic polarization by the tumor UPR. We also show that exogenous IL-2, added either during cross-priming or during restimulation by antigen, does not rescue proliferation (Fig. 5B-C), arguing against classical CD4^+^ T cell-type anergy [53]. Proliferation-refractory CD8^+^ T cells could be rescued by addition of either excess SIINFEKL peptide or L-norvaline during cross-priming (Fig. 5A,D), suggesting that the proliferative defect may be the result of insufficient ligation of the TCR, suppression due metabolite depletion (e.g., arginine), or both. Based on these considerations, and the fact that CD8^+^ T cells cross-primed by TERS-imprinted myeloid DC produce IL-2 (Fig. 6C), we propose that the type of proliferation refractoriness described here is also different from division arrest anergy described in CD4^+^ T cells [54] or from activation-induced unresponsiveness described in CD8^+^ T cells that have lost their ability to make IL-2 [55]. Collectively, the data show that proliferation-refractory CD8^+^ T cells described here represents a hitherto unknown type of CD8^+^ T cell unresponsiveness, downstream of transmission of ER stress from tumor cells to myeloid DC as the putative causative mechanism.

Proliferation-refractory CD8^+^ T cells possess an interesting transcriptional profile with upregulation of *Il-10*, *Tnf-*α and FOXP3, which together with the down-regulation of CD28 suggest an incipient plastic differentiation toward a regulatory phenotype. Several examples in the literature suggest that the CD8^+^ T cells resulting from cross-priming by TERS-imprinted myeloid DC described here may be regulatory/suppressive in nature. For instance, human tumor-infiltrating CD8^+^/CD28^-^ regulatory T cells secrete IL-10, TNF-α, and IFN-γ, express FOXP3, and suppress the proliferation of CD8^+^ effector T cells [56], [57]. Furthermore, ER stress has been associated with the differentiation of human CD4^+^ and CD8^+^ T cells into FOXP3^+^/IL-10-producing regulatory T cells [58]. Since our present analysis examined the initial effector phase of cross-priming, and the T cells display several of the characteristics of human regulatory CD8^+^ T cells, future studies will need to assess their long-term fate as well as their role in regulating anti-tumor immunity *in vivo*.

An unexpected finding of our study is the upregulation of the spliced form of *Xbp-1* (Fig. 6A). The IRE1-XBP-1 branch of the UPR has previously been shown to be critically involved in plasma cell differentiation [59], and DC development [60], but little is still known about its role in T cells. Gene profiling studies showed that XBP-1 mRNA is up-regulated by IL-2 in CD4 T cells [61]. A recent report showed that *Xbp-1* is only transiently (<24 h) spliced in CD8^+^ T cells after anti-CD3 and anti-CD28 antibody stimulation [62]. In contrast, we show that *Xbp-1s* remains elevated four days after initial TCR ligation raising questions about the role of the IRE1-XBP-1 axis in restraining T cell proliferation and shaping CD8^+^ T cell fate during cross-priming. During normal CD8^+^ T cell activation, *Xbp-1* splicing may represent a compensatory mechanism that enables T cell survival in anticipation of pending needs for protein synthesis and processing such as the synthesis and release of lytic enzymes and cytokines. In agreement with this interpretation, a recent report in *Caenorhabditis elegans* [63] showed that XBP-1 upregulation is a compensatory mechanism required to recover from and survive the stress associated with the elicitation of an innate immune response. Thus, we provisionally conclude that the protracted upregulation of *Xbp-1s* in CD8^+^ T cells cross-primed by TERS-imprinted myeloid DC serves to keep the cell in a state of functional readiness until the state of proliferation refractoriness wanes. It cannot be excluded that prolonged inactivation of proliferation may promote conversion to a T_reg_ cell fate as suggested [64]. The implications of prolonged activation of the IRE1-XBP1 axis on cell fate and function of the CD8^+^ T cells will need to be elucidated.

### Conclusions

In conclusion, we demonstrated the cell-extrinsic role of the tumor UPR in polarizing myeloid DC to a mixed pro-inflammatory/suppressive phenotype, which is hallmarked by rapid maturation, the loss of the ability to cross-present and cross-prime model antigen to naïve CD8^+^ T cells, and the facilitation of tumor growth *in vivo*. Our findings substantiate the proposal that several of the immune defects observed in the tumor microenvironment may be due to the cell-extrinsic effects of the tumor UPR [11]. As the tumor UPR is also a crucial cell-intrinsic mechanism of tumor survival *in vivo* [6], [9], new therapies targeting the UPR could offer a two-fold benefit: retardation of tumor cell adaptation and growth [65], and reversal of tumor-induced immune suppression and tolerance, which presently stand as barriers to immunotherapy and autochthonous anti-tumor T cell responses.

## Supporting Information

Figure S1 **TERS-imprinted BMDC exhibit an activated, mature morphology.** BMDC were cultured for 24 hrs in TERS^cm^ or Veh^cm^ from the tumor cell lines indicated and photographed under 20X objective. Results are representative of at least three independent experiments.
(TIFF)

Figure S2 **CD8^+^ T cells cross-primed by TERS-imprinted BMDC without antigen do not proliferate nor become activated.** BMDC were cultured in TERS^cm^ or Veh^cm^ from B16.F10 tumor cells, or media alone (Unstim) for 8 hrs after which OVA (1 mg/mL) was (+OVA) or was not (-OVA) added directly to cultures for a further 16 hrs. BMDC were then co-cultured with CFDA-SE-labeled CD8^+^ OT-I transgenic T cells. After 96-hr co-culture, CD8^+^ T cells were interrogated for (**A**) proliferation (CFDA-SE dilution) and (**B**) expression of the indicated activation markers, by flow cytometry. Results are representative of two experiments.
(TIFF)

Figure S3 **CD8^+^ T cells cross-primed by TERS-imprinted BMDC do not upregulate PD-1.** BMDC were cultured in TERS^cm^ or Veh^cm^ from B16.F10 tumor cells, or media alone (Unstim) for 8 hrs after which OVA (1 mg/mL) was added directly to cultures for a further 16 hrs. BMDC were then co-cultured with CFDA-SE-labeled or unlabeled CD8^+^ OT-I transgenic T cells. After 96-hr co-culture, CD8^+^ T cells were interrogated for (**A**) CFDA-SE dilution and PD-1 expression, or (**B**) PD-1 expression alone by flow cytometry. Results are representative of three experiments.
(TIFF)

Figure S4 **CD8^+^ T cells cross-primed by TERS-imprinted BMDC proliferate after removal from co-culture.** BMDC were cultured in TERS^cm^ from B16.F10 tumor cells or media alone (Unstim) for 8 hrs after which ovalbumin (1 mg/mL) was added directly to cultures for a further 16 hrs. BMDC were then co-cultured with CFDA-SE-labeled CD8^+^ OT-I transgenic T cells. After 96-hr co-culture, CD8^+^ T cells were recovered, cultured without antigen for 96 hrs, and assessed for proliferation by CFDA-SE dilution.
(TIFF)

Figure S5 **The dominant suppressive activity of TERS-imprinted BMDC is not rescued by arginase inhibition.** BMDC were cultured in TERS^cm^ or Veh^cm^ from B16.F10 tumor cells, or media alone (Unstim) for 8 hrs after which OVA (1 mg/mL) was (+OVA) or was not (-OVA) added directly to cultures for a further 16 hrs. OVA-fed Unstim BMDC were co-cultured with TERS-imprinted BMDC, with or without antigen, and CFDA-SE-labeled CD8^+^ OT-I T cells with or without L-nor (10 mM). After 96-hr co-culture, CD8^+^ T cells were interrogated for proliferation by CFDA-SE dilution by flow cytometry. Results are representative of two independent experiments.
(TIFF)
