# Supplementary figures and images for: Cell-Extrinsic Effects of Tumor ER Stress Imprint Myeloid Dendritic Cells and Impair CD8+ T Cell Priming
Source: PLoS One. 2012 Dec 18;7(12):e51845. doi: 10.1371/journal.pone.0051845 (PMC3525659; doi:10.1371/journal.pone.0051845)

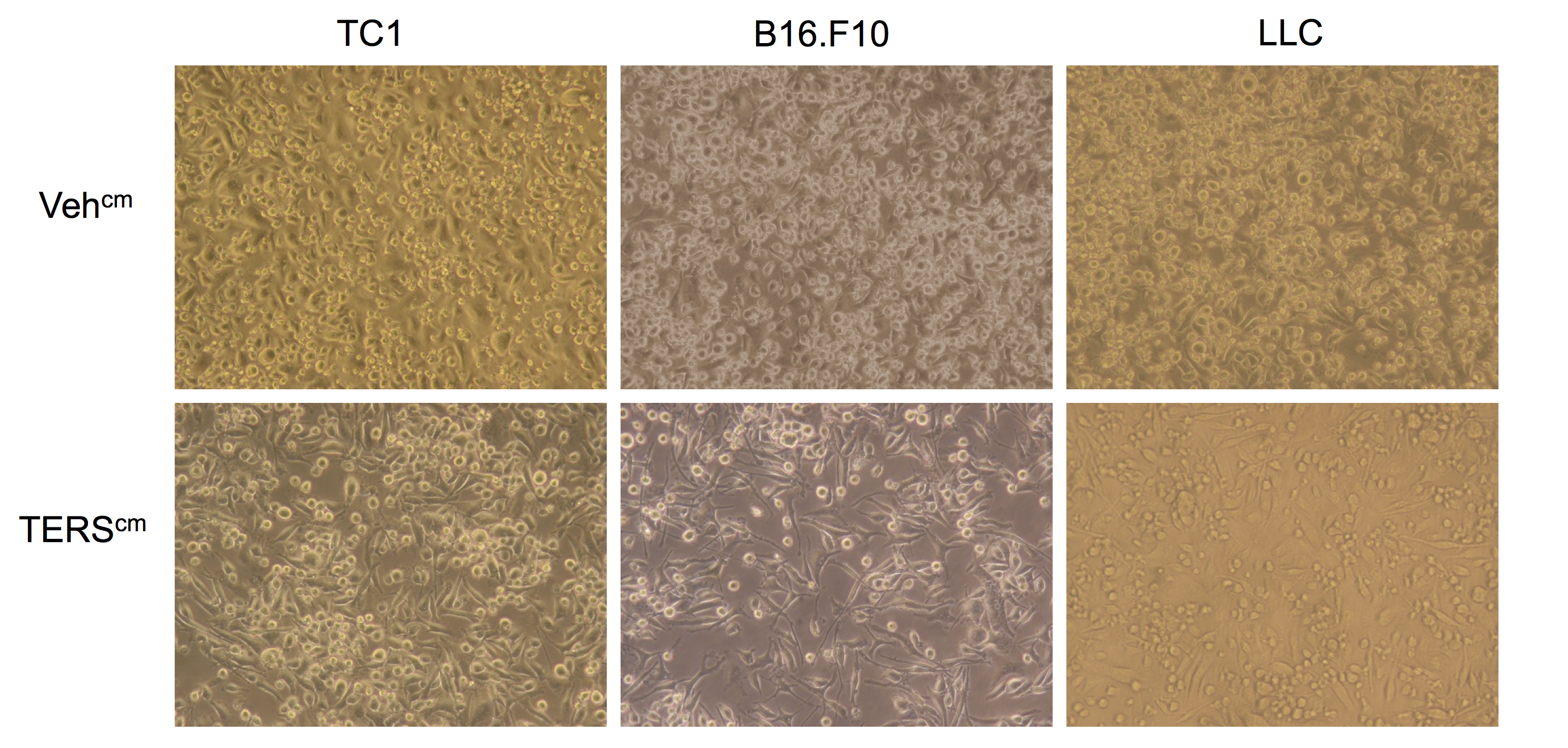

Supplement: Figure S1 — TERS-imprinted BMDC exhibit an activated, mature morphology. BMDC were cultured for 24 hrs in TERScm or Vehcm from the tumor cell lines indicated and photographed under 20X objective. Results are representative of at least three independent experiments. (TIFF) [file pone.0051845.s001.tiff]

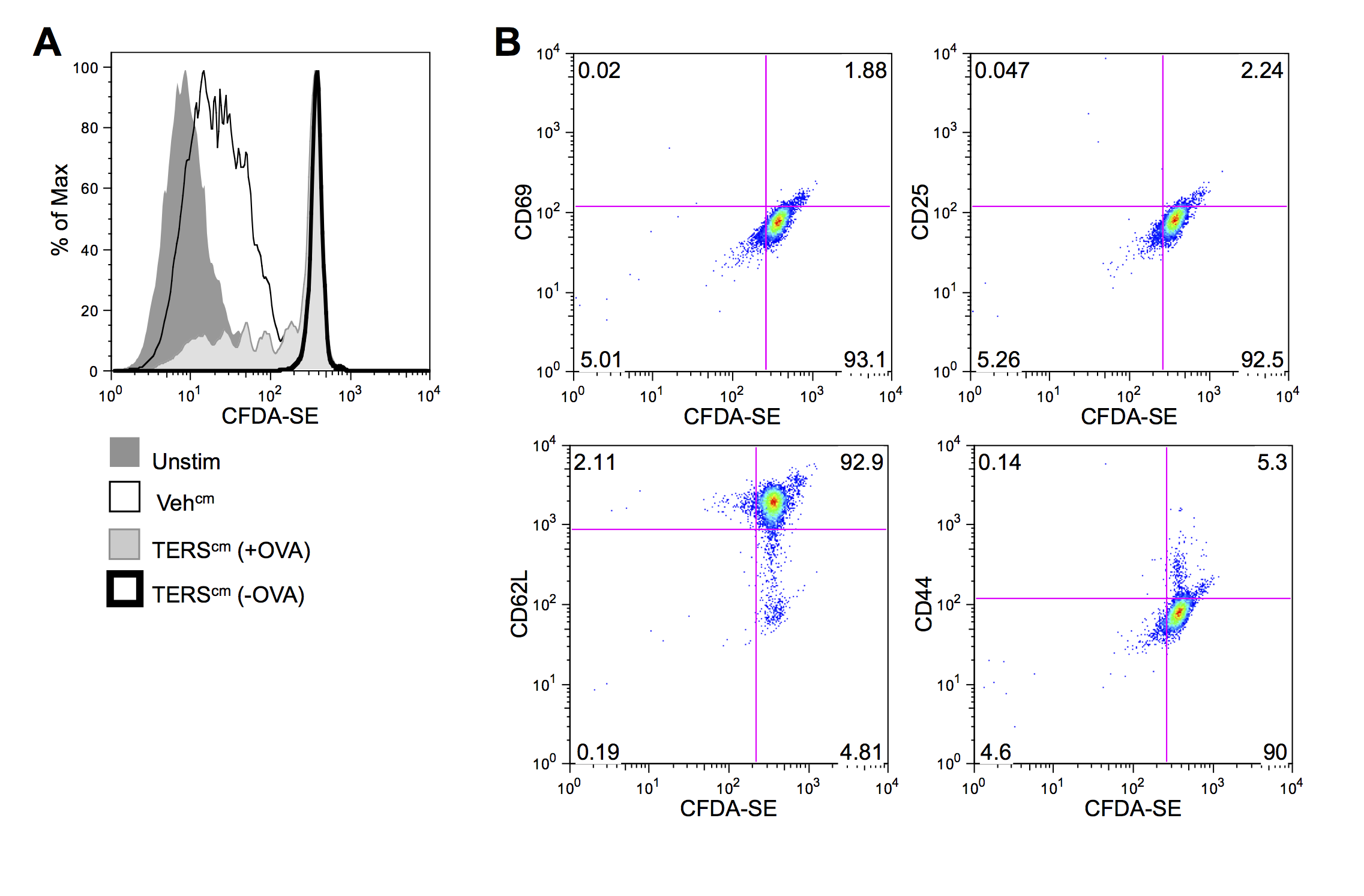

Supplement: Figure S2 — CD8+ T cells cross-primed by TERS-imprinted BMDC without antigen do not proliferate nor become activated. BMDC were cultured in TERScm or Vehcm from B16.F10 tumor cells, or media alone (Unstim) for 8 hrs after which OVA (1 mg/mL) was (+OVA) or was not (-OVA) added directly to cultures for a further 16 hrs. BMDC were then co-cultured with CFDA-SE-labeled CD8+ OT-I transgenic T cells. After 96-hr co-culture, CD8+ T cells were interrogated for (A) proliferation (CFDA-SE dilution) and (B) expression of the indicated activation markers, by flow cytometry. Results are representative of two experiments. (TIFF) [file pone.0051845.s002.tiff]

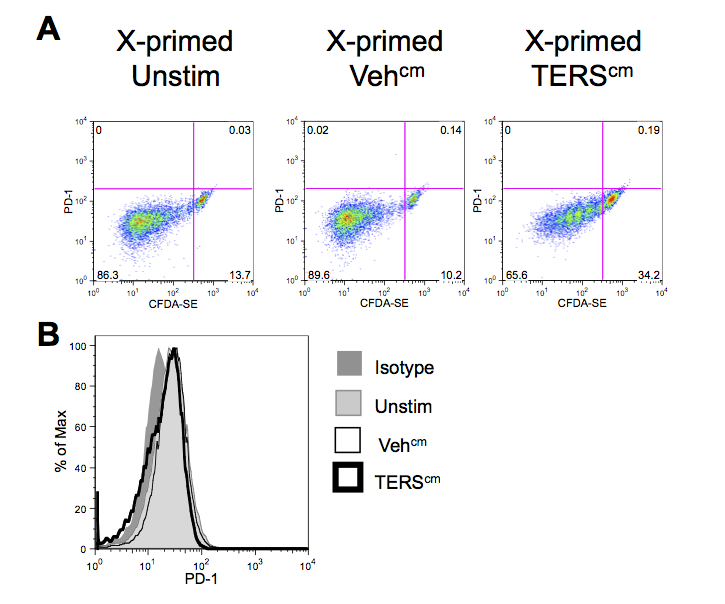

Supplement: Figure S3 — CD8+ T cells cross-primed by TERS-imprinted BMDC do not upregulate PD-1. BMDC were cultured in TERScm or Vehcm from B16.F10 tumor cells, or media alone (Unstim) for 8 hrs after which OVA (1 mg/mL) was added directly to cultures for a further 16 hrs. BMDC were then co-cultured with CFDA-SE-labeled or unlabeled CD8+ OT-I transgenic T cells. After 96-hr co-culture, CD8+ T cells were interrogated for (A) CFDA-SE dilution and PD-1 expression, or (B) PD-1 expression alone by flow cytometry. Results are representative of three experiments. (TIFF) [file pone.0051845.s003.tiff]

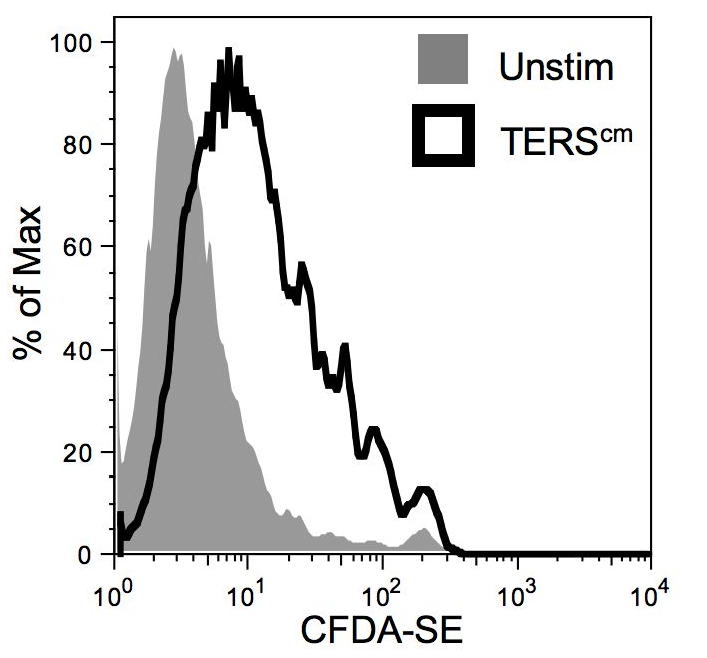

Supplement: Figure S4 — CD8+ T cells cross-primed by TERS-imprinted BMDC proliferate after removal from co-culture. BMDC were cultured in TERScm from B16.F10 tumor cells or media alone (Unstim) for 8 hrs after which ovalbumin (1 mg/mL) was added directly to cultures for a further 16 hrs. BMDC were then co-cultured with CFDA-SE-labeled CD8+ OT-I transgenic T cells. After 96-hr co-culture, CD8+ T cells were recovered, cultured without antigen for 96 hrs, and assessed for proliferation by CFDA-SE dilution. (TIFF) [file pone.0051845.s004.tiff]

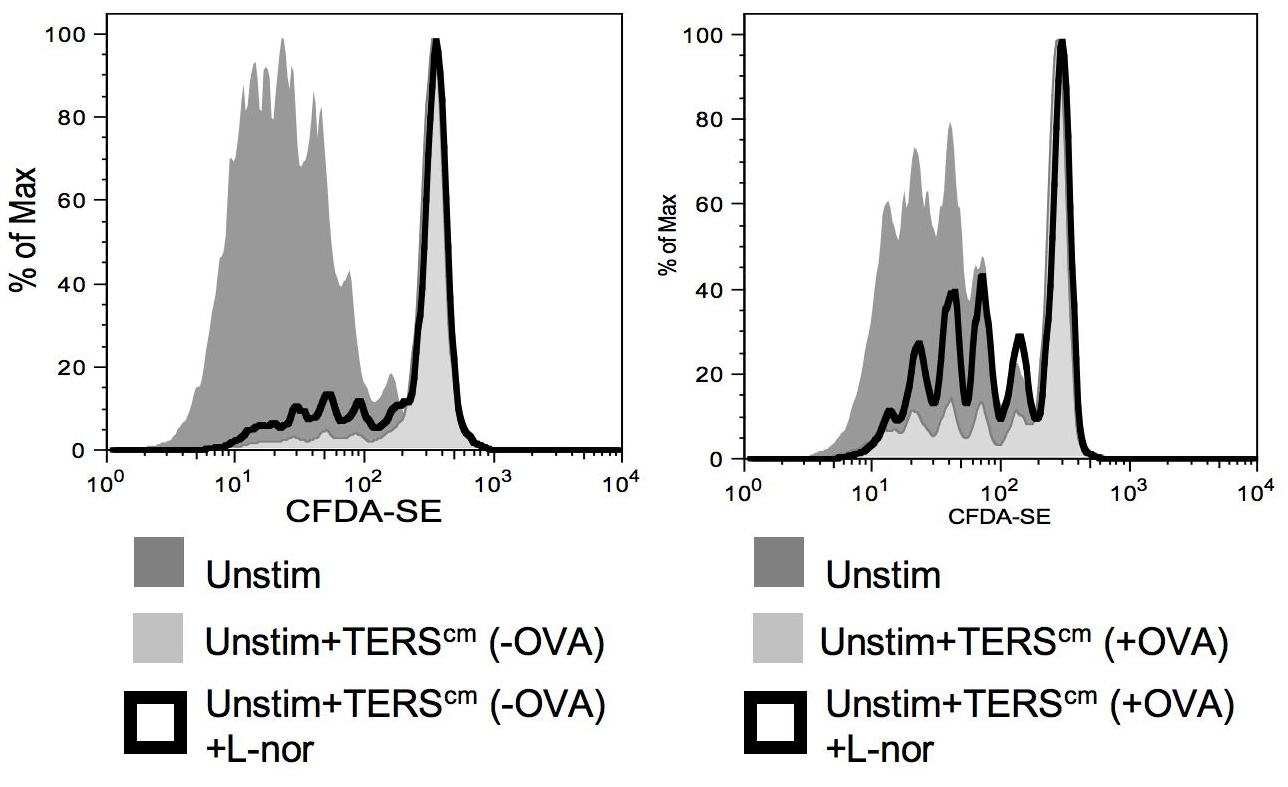

Supplement: Figure S5 — The dominant suppressive activity of TERS-imprinted BMDC is not rescued by arginase inhibition. BMDC were cultured in TERScm or Vehcm from B16.F10 tumor cells, or media alone (Unstim) for 8 hrs after which OVA (1 mg/mL) was (+OVA) or was not (-OVA) added directly to cultures for a further 16 hrs. OVA-fed Unstim BMDC were co-cultured with TERS-imprinted BMDC, with or without antigen, and CFDA-SE-labeled CD8+ OT-I T cells with or without L-nor (10 mM). After 96-hr co-culture, CD8+ T cells were interrogated for proliferation by CFDA-SE dilution by flow cytometry. Results are representative of two independent experiments. (TIFF) [file pone.0051845.s005.tiff]
